# Overexpression of cyclic GMP-dependent protein kinase reduces MeCP2 and HDAC2 expression

**DOI:** 10.1002/brb3.92

**Published:** 2012-09-17

**Authors:** Elodie Deschatrettes, Peggy Jouvert, Jean Zwiller

**Affiliations:** Laboratoire d'Imagerie et de Neurosciences Cognitives, UMR 7237 CNRS, Université de StrasbourgStrasbourg, France

**Keywords:** cGMP-dependent protein kinase, cocaine, cyclic GMP, DNA methylation, histone deacetylase, methyl-CpG-binding protein MeCP2

## Abstract

Nitric oxide (NO) and the C-type natriuretic peptide (CNP) exert their action via stimulation of the cyclic GMP (cGMP)-signaling pathway, which includes the activation of cGMP-dependent protein kinases (PKG). The present report shows that the activation of PKG by local application of 8-bromo-cGMP in the caudate–putamen reduced the expression of the epigenetic markers, methyl-CpG-binding protein 2 (MeCP2) and histone deacetylase 2 (HDAC2), in dopaminergic projection areas of cocaine-treated rats. An effect of lesser amplitude was observed when rats were not injected with cocaine. We also studied the effect of PKG overexpression by injecting a plasmid vector containing the human PKG-Iα cDNA in either the caudate–putamen or the ventral tegmental area. Injection in the caudate–putamen reduced the epigenetic parameters with higher amplitude than the cGMP analog. The effect was abolished by the injection of a selective PKG inhibitor, confirming that it was due to PKG-dependent phosphorylation. As MeCP2 and HDAC2 modulate dynamic functions in the adult brain such as memory formation and synaptic plasticity, the downregulation of expression by PKG suggests that the cGMP pathway affects cognitive processes through a mechanism that comprises the MeCP2/HDAC2 complex and the subsequent control of gene silencing.

## Introduction

Guanosine 3′,5′-cyclic monophosphate (cGMP) is an intracellular second messenger that is synthesized in response to the activation of either soluble guanylyl cyclase by nitric oxide (NO) (Miki et al. 1977; Chinkers and Garbers 1991) or the membrane-bound guanylyl cyclase GC-B primarily by the C-type natriuretic peptide (CNP) (Potter et al. 2006). Cyclic GMP effects are predominantly mediated by the activation of cGMP-dependent protein kinases (PKGs). Two distinct mammalian PKGs, PKG-I and PKG-II, have been identified, as well as two splice variants of PKG-I (PKG-Iα and -Iβ). Both PKG-I and -II are found in the brain; PKG-I is highly expressed in cerebellar Purkinje cells and, to a lesser extent, in striatal medium-spiny neurons (Lohmann et al. 1981; Ariano 1983; DeCamilli et al. 1984; Jarchau et al. 1994). PKGs are implicated in various aspects of brain physiology, including development (Guan et al. 2009), neurotransmitter release (Guevara-Guzman et al. 1994; Lin et al. 1995), and synaptic plasticity (Zhuo et al. 1994).

Alteration of gene expression in response to drugs of abuse is thought to underlie the persistent behavioral changes associated with chronic use (Nestler 2001). Epigenetic mechanisms that regulate the accessibility of genes to the transcriptional machinery in the mature brain control changes in gene expression produced by cocaine (Colvis et al. 2005; Cassel et al. 2006). In the nucleosome, DNA methylation and posttranslational modifications of histones are the major epigenetic mechanisms. Histone acetylation on lysine residues in the amino-terminal tail is the most frequent posttranslational histone modification. In general, increased histone acetylation leads to DNA relaxation and elevated transcriptional activity, whereas decreased acetylation brought about by histone deacetylases (HDACs) results in tighter DNA coiling and gene silencing (Jenuwein and Allis 2001; De Ruijter et al. 2003). We have shown that repeated administration of cocaine induced the expression of the methylated DNA-binding protein, methyl-CpG-binding protein 2 (MeCP2), therefore increasing HDAC activity (Cassel et al. 2006). MeCP2 expression and histone acetylation have also been implicated in long-term changes produced by cocaine self-administration (Host et al. 2011) and nicotine place preference (Pastor et al. 2011) in rats.

In previous studies, we have shown that activation of the cGMP pathway attenuated several effects of cocaine. Intracerebroventricular application of CNP reduced the increase in synaptic dopamine and immediate early gene expression produced by cocaine (Thiriet et al. 2001). CNP also decreased alcohol intake when injected directly into dopaminergic brain areas (Romieu et al. 2008). Moreover, a similar inhibitory effect on cocaine-induced *egr-1* expression and locomotor activity was obtained by simply activating or overexpressing PKG-I in dopaminergic structures, using the polyethyleneimine delivery system (Jouvert et al. 2004). The delivery protocol produced an active enzyme with the expected relative molecular weight of 75,000 and the highest kinase activity was observed 24 h after plasmid delivery. When the PKG plasmid was injected into the ventral tegmental area (VTA), it was expressed in dopaminergic neurons, and injection into the caudate–putamen (CPu) produced an overexpression of the kinase in medium-spiny neurons, that use γ-aminobutyric acid (GABA) as neurotransmitter, and in cholinergic and GABAergic interneurons (Jouvert et al. 2004). In this study, we investigated whether activating the cGMP pathway would also influence the expression of the epigenetic parameters, MeCP2 and HDAC2.

## Methods

### Animals

Male Wistar rats (Janvier, France), weighing 220–270 g, were housed in standard cages under a fixed 12 h light/dark cycle (lights on at 07:00 am) with ad libitum access to food and water. All procedures involving animal care were conducted in compliance with current laws and policies (Council directive 87848, *Service Vétérinaire de la Santé et de la Protection Animales*; permission 67-165 to J. Z.). For implantation of guide cannulae (external diameter, 0.6 mm; internal diameter, 0.4 mm; Plastics One, Roanoke, VA), rats were anesthetized with ketamine (100 mg/kg). A guide cannula was stereotaxically implanted into either the CPu (0.3 mm anterior to bregma; 3.5 mm lateral to bregma; 4.8 mm ventral from the skull) or the VTA (2.3 mm anterior to lambda; 0.5 mm lateral to bregma; 7.7 mm ventral to skull surface) (Paxinos and Watson 1997). The guide cannula was permanently fixed to the skull with stainless steel screws and methacrylate cement. Experiments were performed 4 days after implantation. The correct placement of guide cannulae was verified by histological examination of tissue sections. Data from rats in which placements were not localized in the intended site were not included in any analyses.

### PKG activation and overexpression

**Figure 1:**
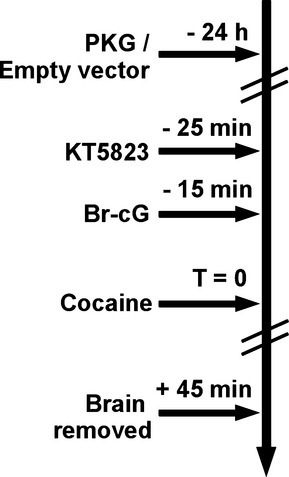
Schematic representation of the injection schedule used for PKG overexpression. Rats were given injections of a vector containing the PKG-I cDNA (0.5 μg) into the right VTA (0.6 μL) or CPu (2 μL) and of 0.5 μg of vector lacking a cDNA insert into the left corresponding structure. Twenty-four hours later, they were injected bilaterally with 2 nmol of KT5823 or saline followed 10 min later by injections of 20 nmol of Br-cG or saline into the same structures. Injection volumes were 0.5 μL for VTA and 1 μL for CPu. Finally, they received an intraperitoneal injection of 20 mg/kg cocaine. Rats were killed and the brains removed 45 min after cocaine injection.

For PKG overexpression, plasmid/polyethyleneimine (PEI) complexes were injected into the CPu (2 μL) or VTA (0.6 μL), as indicated above. After being complexed with PEI in 5% glucose solution, a p513 vector (7.5 μg) containing the human PKG-Iα cDNA and vector lacking a cDNA insert were injected into the right hemi-structure and left hemi-structure, respectively, as described previously (Jouvert et al. 2004). PKG was activated by the subsequent injection of a 20 mmol/L 8-bromo-cyclic GMP (Br-cG; Sigma-Aldrich, St Louis, Missouri) in a saline solution, or inhibited by the injection under identical conditions of a 2 mmol/L KT5823 (Calbiochem, Merck, Darmstadt, Germany) solution, according to the schedule shown in Figure 1. The injection volume was 0.5 μL for the VTA and 1 μL for the CPu. Control microinjections were equivalent volumes of vehicle. Cocaine (Cooper, Melun, France) was injected intraperitoneally at the dose of 20 mg/kg.

### Immunohistochemistry

Animals were given an overdose of pentobarbital (100 mg/kg, intraperitoneally) 45 min following cocaine injection and were then perfused transcardially with 100 mL saline followed by 2% paraformaldehyde in phosphate-buffered saline (PBS; 0.1 mol/L; pH 7.2; 250 mL). The brains were removed, kept overnight at 4°C in 15% sucrose, frozen in isopentane at −40°C, and stored at −80°C. Coronal sections (20 μm thick) were obtained using a Microm HM560 cryostat.

Immunohistochemistry was performed as described previously (Cassel et al. 2006). Briefly, brain sections were incubated with the following primary polyclonal antibodies: anti-MeCP2 (1:150 dilution; Millipore, Billerica, MA) or anti-HDAC2 (1:200 dilution, Millipore). Sections were then successively incubated with biotinylated donkey anti-rabbit IgG (1:200 dilution) and with an avidin–biotin–peroxidase complex (Vectastain ABC kit, Vector Laboratories, Burlingame, CA). Staining was revealed with the chromagen 3,3′-diaminobenzidine tetrahydrochloride and H_2_O_2_. Sections were then incubated in a 2.5 μmol/L bisbenzimide (Hoechst 33258; Sigma-Aldrich) solution to label nuclei and the slides were coverslipped with Mowiol. Staining was observed under a fluorescent Leitz DM RB binocular microscope (Leica Microsystems, Wetzlar, Germany). Two photomicrographs of the same field were taken with an Axiocam camera (Carl Zeiss, Iena, Germany); one was used to count the total number of nuclei stained with Hoechst 33258 and the other to count the number of immunoreactive cells for a given antigen. Positively stained nuclei or cells were counted, using the plugin Cell Counter tool of ImageJ 1.43 software (NIH, MA). The percentage of immunoreactive cells was calculated from counts on at least 800 cells by an investigator blind to the experimental conditions. For each measure, 6–8 counts were performed on four sections from 3 to 6 different rats.

### Statistical analysis

Results are expressed as means ± SEM. Protein expression was analyzed by a one-way analysis of variance (ANOVA) on the data from each treatment. Student–Newman–Keuls post hoc tests were performed when required, and significance was set at *P* ≤ 0.05. Statistical analysis was performed using SigmaStat (Systat software, Chicago, IL).

## Results

### Effect of PKG activation and overexpression on MeCP2 expression in cocaine-treated rats

**Figure 2:**
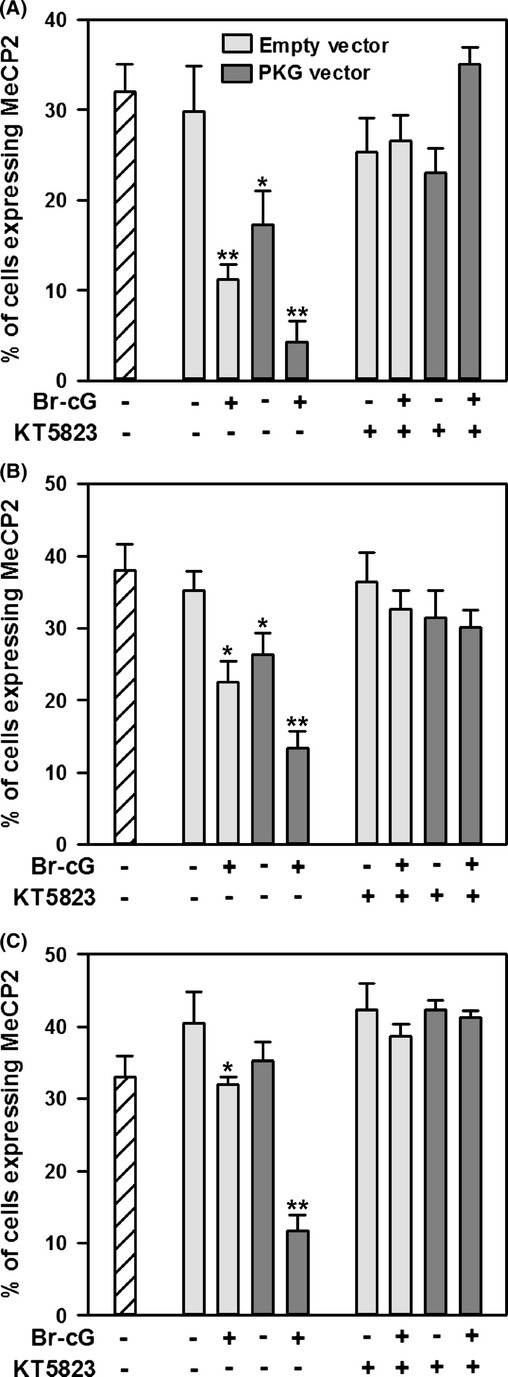
Quantification of cells expressing MeCP2 in response to the activation or overexpression of PKG in the CPu. Quantification was carried out in (A) the dorsal CPu, (B) the NAc shell, and (C) the PFCx (*n* = 3 rats in the groups that were injected with KT5823 and *n* = 6 in the other groups). Treatment of rats and immunohistochemistry were performed as described under Methods and in Figure 1. Hatched bars on the left represent the values obtained in control rats that were not treated with cocaine. Nuclei were stained with bisbenzimide (Hoechst 33258). The percentage of immunoreactive cells was calculated from 6 to 8 counts on three serial sections. *P < 0.05, **P < 0.001, comparison with control rats injected with the empty vector and saline (ANOVA followed by Student–Newman–Keuls post hoc test).

The effect of PKG activation on MeCP2 expression was studied by injecting Br-cG, a cell permeant analog of cGMP, into the CPu or the VTA, according to the protocol described in Figure 1. Previous studies have shown that a 15-min period was sufficient to optimally activate the kinase enzymatic activity. The inhibitor was injected 10 min before the activator, to ensure that the enzyme was in an inhibited state before injection of the activator. Quantitative analysis of cells expressing MeCP2 in the dorsal CPu, in the shell subregion of the nucleus accumbens (NAc), and in the prefrontal cortex (PFCx) in response to intra-CPu injection of Br-cG is shown in Figure 2. Acute cocaine treatment did not significantly increase MeCP2 expression. Activation of PKG by Br-cG microinjection into the CPu caused a 63% decrease in MeCP2 levels in the dorsal CPu. A smaller decrease was found in the NAc shell (32%) and in the PFCx (21%) under the same conditions.

**Figure 3:**
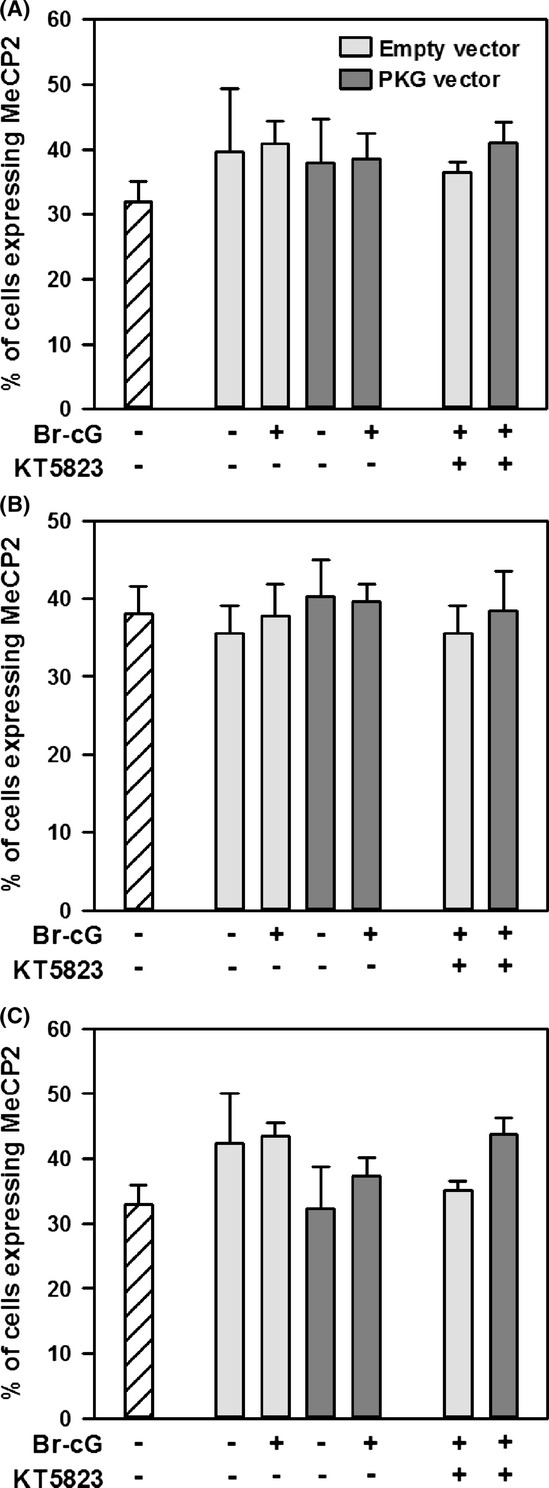
Quantification of cells expressing MeCP2 in response to the activation or overexpression of PKG in the VTA. Quantification was carried out in (A) the dorsal CPu, (B) the NAc shell, and (C) the PFCx (*n* = 3 in the groups that were injected with KT5823 and *n* = 6 in the other groups). Hatched bars on the left represent the values obtained in control rats that were not treated with cocaine. The percentage of immunoreactive cells was calculated from 5 to 7 counts on three serial sections.

The effect of PKG overexpression on MeCP2 expression was studied after injection of the PKG plasmid into the same site than that used for Br-cG injection, according to the protocol described in Figure 1. In the CPu, the overexpression of the kinase by itself reduced MeCP2 levels by 42%; full activation of the exogenous kinase by Br-cG further reduced MeCP2 expression to a very low level. The effect was less pronounced in the two other structures (Fig. 2). All these effects were blocked by the prior injection of KT5823, a selective inhibitor of PKG. Figure 3 shows quantitative analysis of MeCP2 expression in the CPu, NAc, and PFCx in response to intra-VTA injections. Surprisingly, MeCP2 expression was not significantly reduced in any of the structures even though PKG overexpression produced a decrease in the PFCx.

### Effect of PKG activation and overexpression on HDAC2 expression in cocaine-treated rats

**Figure 4:**
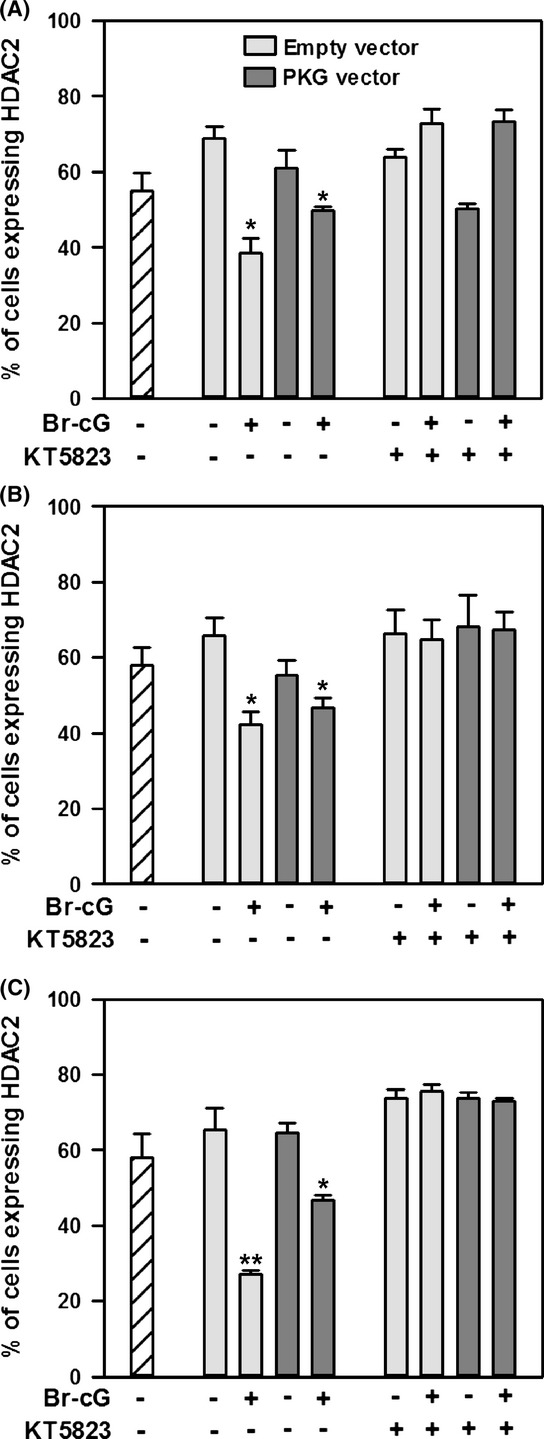
Quantification of cells expressing HDAC2 in response to the activation or overexpression of PKG in the CPu. Quantification was carried out in (A) the dorsal CPu, (B) the NAc shell, and (C) the PFCx (*n* = 3 in the groups that were injected with KT5823 and *n* = 6 in the other groups). Hatched bars on the left represent the values obtained in control rats that were not treated with cocaine. The percentage of immunoreactive cells was calculated from 6 to 8 counts on three serial sections. *P < 0.05, **P < 0.001, comparison with control rats injected with the empty vector and saline (ANOVA followed by Student–Newman–Keuls post hoc test).

Quantitative analysis of cells expressing HDAC2 in the dorsal CPu, NAc shell, and PFCx in response to intra-CPu injection of the plasmids and of Br-cG is shown in Figure 4. Data were obtained from immunohistochemistry experiments of coronal sections adjacent to those taken for measuring MeCP2 levels. As was the case for MeCP2, HDAC2 levels were not modified by the cocaine injection. When Br-cG was microinjected into the CPu, HDAC2 levels were decreased by about 50% in CPu and PFCx and by about 40% in the NAc. The reduction was completely reversed when the selective PKG inhibitor was injected before Br-cG.

**Figure 5:**
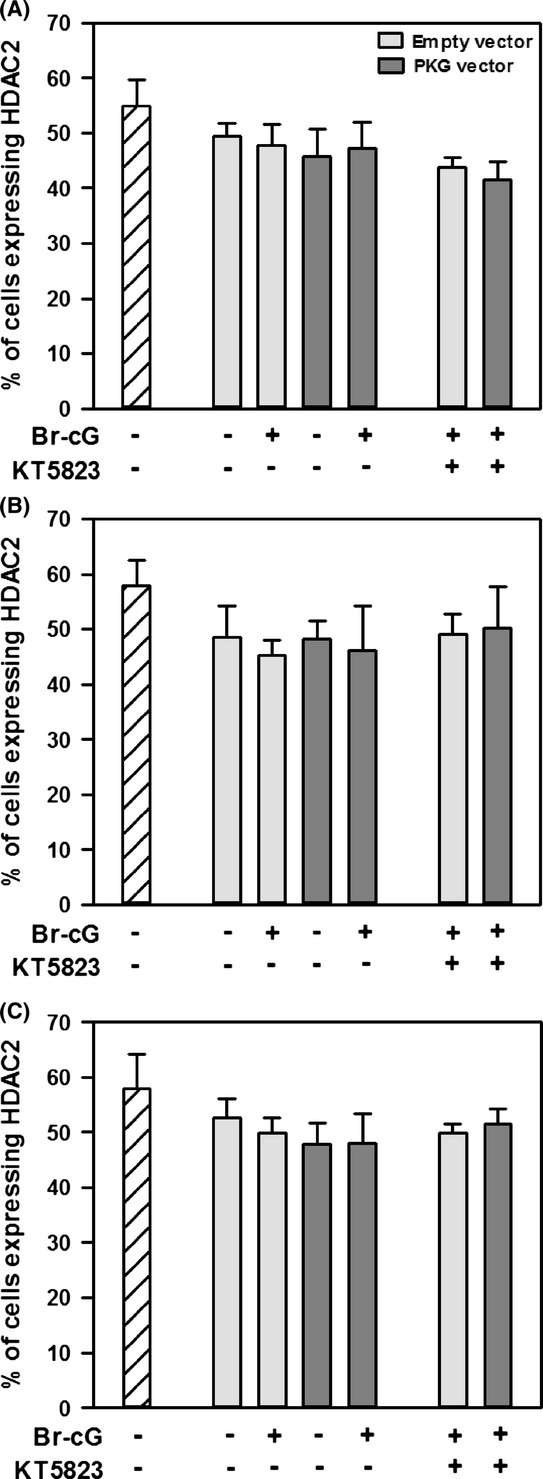
Quantification of cells expressing HDAC2 in response to the activation or overexpression of PKG in the VTA. Quantification was carried out in (A) the dorsal CPu, (B) the NAc shell, and (C) the PFCx (*n* = 3 in the groups that were injected with KT5823 and *n* = 6 in the other groups). Hatched bars on the left represent the values obtained in control rats that were not treated with cocaine. The percentage of immunoreactive cells was calculated from 4 to 7 counts on three serial sections.

In the PFCx, and to a lesser degree in the CPu and NAc, activation by Br-cG of the exogenous kinase resulted in a significant inhibition of HDAC2 expression, but the overexpression of the kinase by itself did not reduce levels of the deacetylase (Fig. 4). Again, the effects of the activation/overexpression were totally blocked by the injection of KT5823. Figure 5 shows quantitative analysis of HDAC2 expression in the dorsal CPu, NAc shell, and PFCx in response to intra-VTA injections. As was the case for MeCP2 expression, HDAC2 levels were not modified.

### Illustration of the PKG effects on MeCP2 and HDAC2 expression

**Figure 6:**
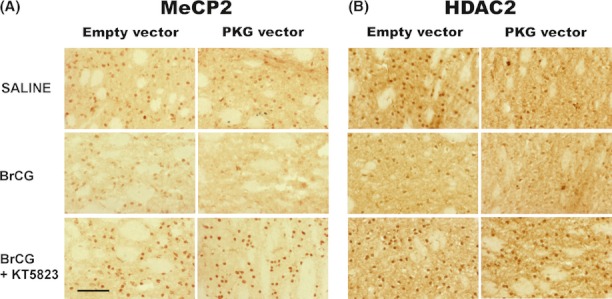
Photomicrographs showing (A) MeCP2 and (B) HDAC2 immunoreactivity in response to the activation or overexpression of PKG in the CPu. Treatment of rats and immunohistochemistry were performed as described under Methods and Figure 1. Coronal tissue sections (20 μm) were incubated with polyclonal antibodies against MeCP2 or HDAC2. Antibody binding was detected with secondary biotinylated antibody and peroxidase reaction. Scale bar applicable to all micrographs, 50 μm.

Figure 6 illustrates MeCP2 and HDAC2 immunostaining in the dorsal CPu of rats that received intra-CPu injections, according to the schedule shown in Figure 1. Both MeCP2 and HDAC2 immunoreactivities were exclusively found in cell nuclei, as expected for proteins that bind DNA or histones. Overexpression of the kinase alone reduced MeCP2 and HDAC2 labeling. Activation of endogenous plus overexpressed kinase reduced the labeling further. The reduction in protein expression was reversed by KT5823, and there was an increase in the number of immunoreactive cells; the labeling intensity of both proteins was somewhat enhanced.

### Effect of PKG activation on MeCP2 and HDAC2 expression in control rats

**Figure 7:**
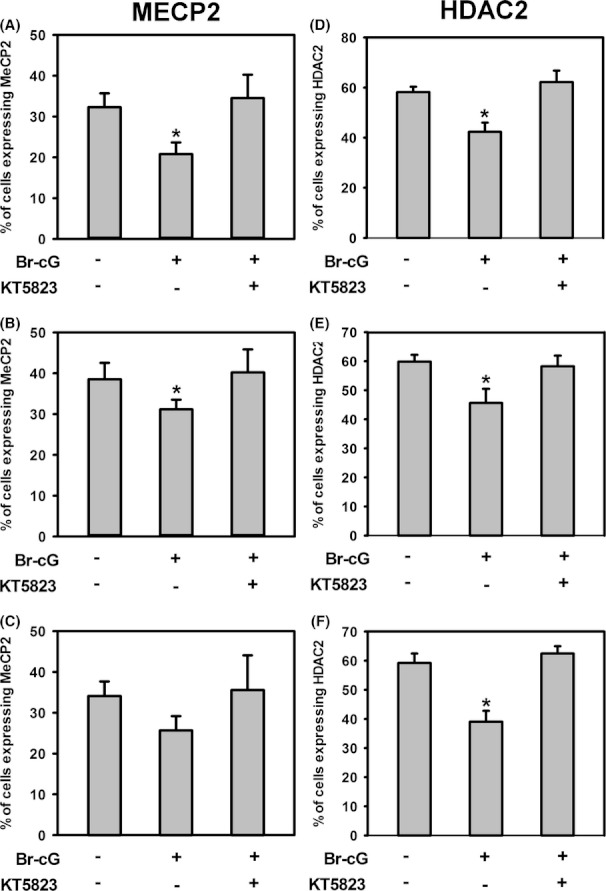
Quantification of cells expressing MeCP2 and HDAC2 in response to the activation of PKG in control rats. Br-cG was injected into the CPu. MeCP2 quantification was carried out in (A) the dorsal CPu, (B) the NAc shell, and (C) the PFCx of rats that were not given further cocaine injection (*n* = 4 per group). HDAC2 quantification was carried out in adjacent sections from (D) the dorsal CPu, (E) the NAc shell, and (F) the PFCx of the same rats. The percentage of immunoreactive cells was calculated from 4 to 6 counts on three serial sections.

We checked whether PKG activation reduced the protein expression also in rats that were not injected with cocaine. Quantitative analysis of cells expressing MeCP2 and HDAC2 in the dorsal CPu, NAc shell, and PFCx in response to intra-CPu injection of Br-cG is shown in Figure 7. Activation of PKG by Br-cG microinjection into the CPu caused a decrease in MeCP2 and HDAC2 levels in all the structures without reaching statistical significance in the PFCx for MeCP2. Overall, a lesser decrease was found when compared with the situation in which rats were given a cocaine injection. These effects were also blocked by the prior injection of the selective PKG inhibitor KT5823.

## Discussion

The present report shows that the activation and/or overexpression of PKG in the CPu strikingly reduced the expression levels of the epigenetic parameters, MeCP2 and HDAC2, in dopaminergic projection areas of cocaine-treated rats. Both proteins were reduced maximally in about 30% of the cells, regardless of the percentage of cells that expressed each protein in control conditions. Studies from several laboratories, including ours, have shown that cocaine-induced modulation in gene expression is achieved, at least partially, via epigenetic regulation (Kumar et al. 2005; Cassel et al. 2006). For instance, DNA methylation and histone acetylation have been implicated in stimulant-related behavioral and molecular adaptations. We found that cocaine increased MeCP2 and HDAC2 nuclear expression, in response to repeated experimenter- or self-administered exposure (Cassel et al. 2006; Host et al. 2011), supporting the hypothesis that epigenetic regulation plays an important role in the development and maintenance of drug addiction. In this study, PKG was found to reduce the expression of these epigenetic factors, suggesting that activators of the cGMP pathway may be used as general pharmacological tools for downregulating the MeCP2/HDAC2 complex. This is comforted by the fact that PKG activation also reduced MeCP2 and HDAC2 expression in rats that were not injected with cocaine, although to a lesser extent.

In previous studies, we showed that activation of the cGMP pathway was sufficient to attenuate the increase in extracellular dopamine, immediate early gene expression, and locomotor activation produced by cocaine (Thiriet et al. 2001; Jouvert et al. 2004). As we used a technique to overexpress the PKG in which highest kinase activity was produced 24 h after plasmid delivery (Jouvert et al. 2004), only effects of an acute injection of cocaine could be determined. Surprisingly, the simple activation of the endogenous PKG in the CPu was sufficient to decrease MeCP2 and HDAC2 protein expression in the dorsal CPu, NAc shell, and PFCx in this short timescale. This may result from a decreased protein expression, possibly due to the phosphorylation, and subsequent inactivation of an element of the transcriptional machinery. Alternatively, it may result from the PKG-induced degradation of the proteins. It is also possible that phosphorylation by PKG of the proteins or of some partner proteins may have induced the formation of alternate complexes that prevented accessibility of MeCP2 and HDAC2 to their respective antibodies. This latter possibility is particularly attractive as both proteins were similarly regulated. The effect on both proteins was abolished by the injection of the selective PKG inhibitor, KT5823, confirming that the effect was due to PKG-dependent phosphorylation.

The effect on MeCP2 expression was greatly potentiated in the three structures examined by the addition and subsequent activation of exogenous overexpressed PKG. Surprisingly, the latter effect was not observed when HDAC2 expression was measured. It is noteworthy that other HDACs, such as HDAC5 or HDAC11, which belong to different classes of histone deacetylases that are also regulated by cocaine (Host et al. 2011), were not modulated by PKG (data not shown), suggesting a unique role of HDAC2 in this process.

In contrast, injection of the PKG vector or Br-cG into the VTA failed to alter MeCP2 or HDAC2 levels in any of the brain sites. The data probably underscore a differential action of PKG at the pre- and postsynaptic levels. Effectively, the observation that only intra-CPu infusions produced the effect probably denotes that it results from a PKG-modified signal transduction pathway taking place in neurons postsynaptic from the dopaminergic neuron. Indeed, in an electron microscopy study, we showed that PKG was localized mainly in postsynaptic sites (data not shown). These findings are consistent with our previous results that also failed to find an effect of intra-VTA infusions of Br-cG on cocaine-induced *egr-1* expression in the CPu (Jouvert et al. 2004).

When the PKG vector was injected into the CPu, overexpression of the kinase was found primarily in GABAergic medium-spiny neurons (Jouvert et al. 2004). Activation of the cGMP pathway in these neurons probably modulates GABA release in many projection areas and therefore disrupts the striato-nigro-thalamic loop. Modulation of neurotransmitter release by cGMP has been demonstrated in many systems; cGMP activators like natriuretic peptides inhibit various secretory responses such as aldosterone and catecholamines (Samson et al. 1993; Babinski et al. 1995), whereas endogenous NO modulates the release of several neurotransmitters, including catecholamines, excitatory and inhibitory amino acids, and serotonin (Guevara-Guzman et al. 1994; Prast and Philippu 2001). Accordingly, disturbance of the striato-nigro-thalamic loop will have an impact on cortical neurons. One of these consequences would be the degradation or downregulation of the MeCP2/HDAC2 complex. The precise mechanism of this proposed effect is currently unknown.

HDAC2 was chosen because it is part of a complex composed also of Sin3A and MeCP2, after the latter binds to methylated DNA. The resulting HDAC activity thus carries transcriptional silencing to the corresponding genes (Yang and Seto 2008). The enzyme is also highly expressed in the mesolimbic pathway (Cassel et al. 2006; Broide et al. 2007). HDAC2 has been reported to regulate memory formation and synapse plasticity in mature neurons (Grissom and Lubin 2009; Guan et al. 2009; Pastor et al. 2011). Similarly, MeCP2 is highly expressed in mature neurons where it is required for modulating dynamic functions of the adult brain and mutations within the gene are known to be associated with Rett syndrome (Nelson et al. 2006; Zhou et al. 2006). The fact that PKG was able to downregulate the expression of both MeCP2 and HDAC2 proteins when injected into the CPu suggests that the cGMP pathway affects cognitive processes through a mechanism that comprises the MeCP2/HDAC2 complex and the gene silencing that it controls. Interestingly, *egr-1* may be one of the genes silenced by this mechanism, as levels of AcH3 and AcH4 were increased in the *egr-1* promoter in HDAC2 KO mice (Guan et al. 2009). The fact that activation of PKG reduced both HDAC2 levels and *egr-1* induction suggests that the MeCP2/HDAC2 complex regulates *egr-1* expression, at least to some extent.

Phosphodiesterases have recently been suggested as potential new targets for cognition enhancement (Reneerkens et al. 2009). Results of this study are consistent with this idea and suggest that amplification of the intracellular availability of the second messenger cGMP by phosphodiesterase inhibitors have therapeutic potential for the treatment of neuropsychiatric disorders involving disturbances of mood, emotion, and cognition, including drug addiction.
